# Down-regulation of toll-like receptor 4 alleviates intestinal ischemia reperfusion injury and acute lung injury in mice

**DOI:** 10.18632/oncotarget.14624

**Published:** 2017-01-13

**Authors:** Qiankun Zhu, Guizhen He, Jie Wang, Yukang Wang, Wei Chen, Tai Guo

**Affiliations:** ^1^ Department of Parenteral and Enteral Nutrition, Peking Union Medical College Hospital, Peking Union Medical College and Chinese Academy of Medical Sciences, Beijing, 100730, China; ^2^ National Institute for the Control of Pharmaceutical and Biological Products, China

**Keywords:** toll-like receptor 4 (TLR4), intestinal ischemia reperfusion (IR), acute lung injury (ALI), inflammatory response, oxidative stress

## Abstract

Intestinal ischemia reperfusion (IR) injury is a critical problem, which can cause intestinal injury locally and acute lung injury (ALI) distally by inflammatory responses and oxidative stress. Toll-like receptor 4 (TLR4) is involved in innate immune and inflammatory responses. This study was to determine whether TLR4 mutant can attenuate intestinal and lung injuries after intestinal IR. Wild type (WT) and TLR4 mutant mice were submitted to intestinal IR by occluding the superior mesenteric artery. Histological assessment of the intestine and the lung were conducted by HE staining. The levels of proinflammatory cytokines, oxidative stress markers, apoptotic index and other mediators were measured. In addition, a 24-hour survival study was performed. Histological assessment showed that intestinal IR caused serious injuries in the intestine and the lung, corroborated by increased proinflammatory cytokines in the circulation. TLR4 mutant suppressed the histological injuries as demonstrated by significantly decreased pathological scores. Consistent with the morphological results, the TLR4 mutant mice exhibited remarkably lowered cytokine expressions in the intestine (TNF-α, IL-6, IL-1β, and NF-κB) and the lung (NO, iNOS, MCP-1, MIP-2, NF-κB, and Caspase-3). ALT and creatinine were also significantly dampened in the liver and kidney, respectively. Furthermore, the survival rate over the course of 24 hours was significantly improved. Collectively, the findings reveal that TLR4 mutant significantly abated the intestinal IR injury and ALI at least in part by alleviating the inflammatory response and oxidative stress.

## INTRODUCTION

Intestinal ischemia reperfusion (IR) injury occurs in a various clinical circumstances including mesenteric artery occlusion, small bowel transplantation, vascular surgery procedures, and trauma [1]. Intestinal IR leads to the activation of the local inflammatory responses and changes of several mediators to induce bacterial translocation out of the gastrointestinal tract [2]. These phenomena contribute substantially to the multiple organ failure and systemic inflammatory responses [3, 4], and can damage distant organs, such as lung [5], heart [6], kidney [7] and liver [8], through circulation of many inflammatory factors. Of these distant organs, the lung, characterized by its high mortality rate of 40% under acute injury, appears to be the earliest remote organ affected by this pathogenic process [9].

Acute lung injury (ALI) can manifest in clinic as acute respiratory distress syndrome that is the main cause of death during intestinal IR injuries [10]. The clinical features for ALI consist of a bilateral chest radiographic infiltrates, a pulmonary artery occlusion pressure less than 18 mm Hg, and the PaO2/FiO2 ratio less than 300 mm Hg accompanied by severe hypoxemia [11]. The evolvement of ALI starts from either a direct or an indirect hit to the pulmonary epithelium and endothelium that causes edema, atelectasis, and fibrosis. Albeit the pathophysiology of ALI after intestinal IR remains complex, it is widely acknowledged that oxidative stress and inflammatory responses are two primary mechanisms leading to ALI [12, 13]. Evidence indicated overproduction of nitric oxide catalyzed by inducible nitric oxide synthase worsens the micro-vascular dysfunction in the lung by oxidative damage [14]. In addition, the reactive oxygen species (ROS) plays an important role in mediating epithelial and endothelial injury in the lung by triggering proinflammatory cytokines such as tumor necrosis factor (TNF) -α, interleukin (IL)-1, and IL-6 [15]. Accordingly, therapies aiming at reducing inflammatory response or oxidative stress are, to some extent, theoretically reasonable to ameliorate the lung damage.

Toll-like receptors (TLRs) are a family of highly conserved germline-encoded pattern recognition receptors that have a regulatory function in innate immune and inflammatory responses [16]. Ligands binding to TLRs can trigger various intracellular kinases systems [17], including the nuclear factor-κB (NF-κB), mitogen activated protein kinases, and c-Jun NH2-terminal kinase, all of which are reported to participate in inflammatory immune pathways and oxidative stress in a variety of gastrointestinal diseases [18–20]. TLR4, a member of the TLR family, recognizes many ligands, such as lipopolysaccharide, a component of gram-negative bacteria, and thus exerts a critical role in organic IR injuries, such as liver [21], spinal cord [22], and brain [23]. Interestingly, TLR4-mutant mice have been recently demonstrated to be resistant to trauma/hemorrhagic shock-induced gut injury [24]. However, to date, there are scarce literatures investigating the effects of TLR4 in the process of intestinal IR-induced ALI. Moreover, the functional mechanism of TLR4 in the intestinal IR-induced ALI remains convoluted. Therefore, this study was conceived to observe whether TLR4 knock down could decrease lung injury in a murine model of intestinal IR-caused ALI through inhibition of inflammation and oxidative stress.

## RESULTS

### Protective effects of TLR4 mutant on the severity of IR injury of the small intestine

**Figure 1:**
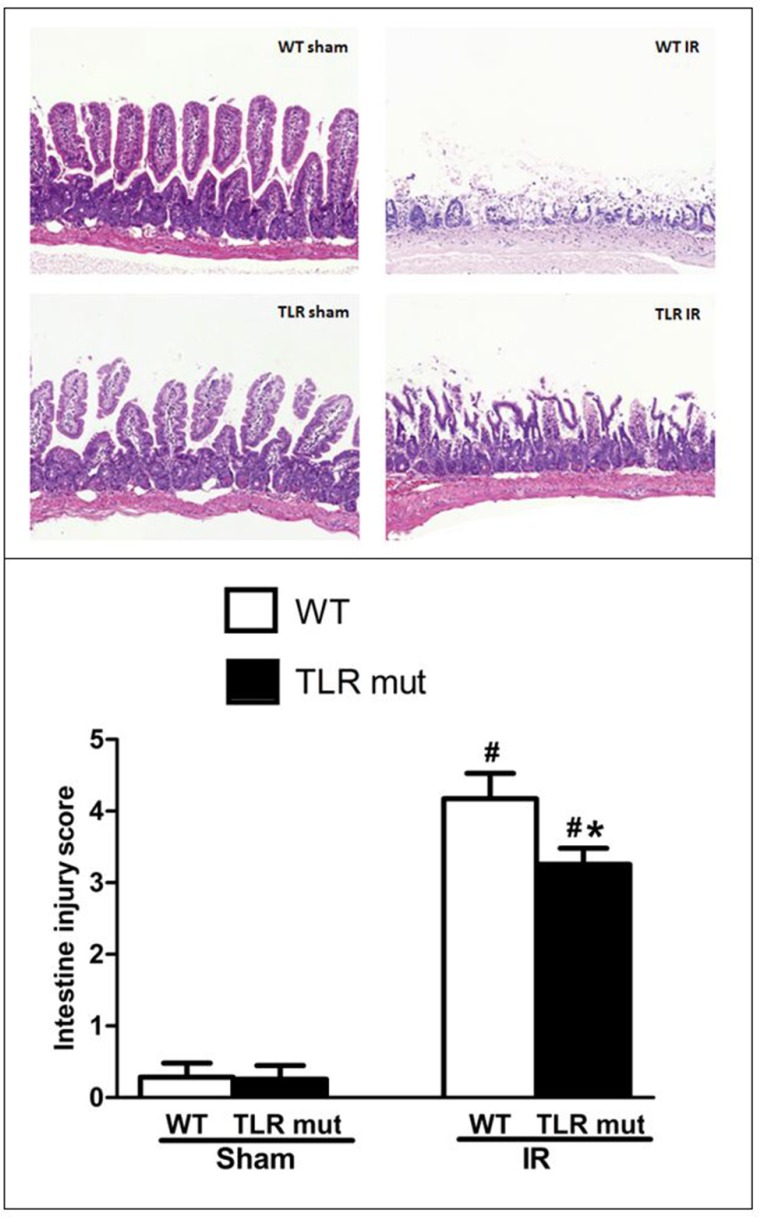
Intestine tissue damage in mice submitted to intestinal IR or sham surgery in each groups TLR4 mutant mice and wild-type (WT) mice were submitted to 60 min of ischemia by blocking the superior mesenteric artery (SMA) and then 120 min of reperfusion. Sham surgery operated the same procedure except for clamping the artery. Representative images of HE staining sections from the WT mice and TLR4 mutant mice are shown (original magnification: X 100). HE staining from the intestine unveiled widespread mucosal damage, loss of villi, and infiltration of inflammatory cells in the WT IR group, while TLR mutant showed beneficial effects. The severity of the intestinal injury was scored by Chiu's grading criteria and each column represents the means ± SD (n = 7/group). Data were compared by Kruskal-Wallis test and Mann-Whitney U test. #*P* < 0.05 vs. WT mice with sham surgery, and *P < 0.05 vs. WT mice with IR.

To determine whether intestinal injury occurred after intestinal IR, HE staining for intestinal histology was evaluated in the first place. In the WT sham groups, no significant pathological injurious indications were observed, the histologic structure of the intestine was integrate, and the denudation of villi could be seen occasionally. However, in the WT mice subjected to IR, histopathologic analysis of the intestine showed severe injury with extensive edema and inflammation. Widespread mucosal destruction and denudation of villi were obvious in the intestine section. These results were reflected in increased pathological scores in the IR group of WT mice, where scores were significantly increased compared with WT sham group (Figure 1).

**Figure 2:**
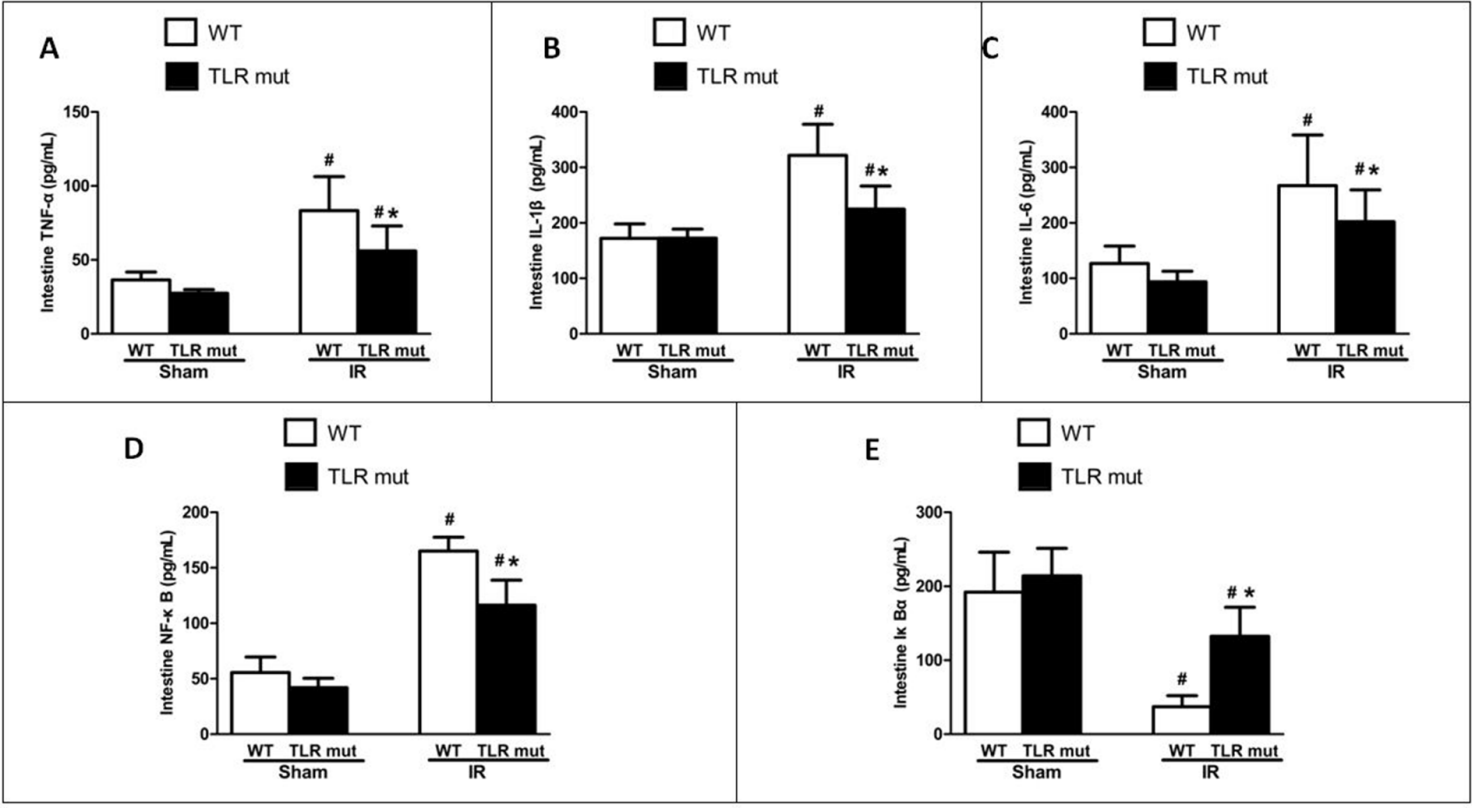
Local cytokine expressions in the intestine after IR WT and TLR4 mutant mice were subjected to intestinal IR or sham surgery. The SMA was clamped for 60 min, followed by 120 min of reperfusion. Intestinal tissue expression of (**A**) TNF-α, (**B**) IL-1β, (**C**) IL-6, (**D**) NF-κB, and (**E**) IκBα were determined 120 min after reperfusion by ELISA. Measurements were in triplicate. Results are given as mean ± SD (n = 7/group). #*P* < 0.05 vs. WT sham and *P < 0.05 vs. WT IR by one-way ANOVA and LSD test.

In the next step, we measured the effects of TLR4 mutant on the intestine after IR injury. As shown in Figure 1, in TLR4 mutant mice submitted to IR, slight pathologic change, a few inflammatory cells, and little loss of villi could be seen. Pathological score of the intestine by HE staining in the TLR4 mutant mice after IR indicated that there was a significant decrement of histological injury score in comparison with the WT IR group, suggesting the protective effect of TLR4 knock-down in the intestinal IR injury (Figure 1). Consistent with the pathological scores, the inflammatory mediators of the intestinal tissue, including TNF-α, IL-6, and IL-1β, were significantly attenuated in the TLR4 mutant IR group than in the WT IR group (Figure 2A–2C). In addition, compared with the WT IR group, the NF-κB, one apoptosis indicator, was presented with significantly lesser expression in the TLR4 mutant IR group, while the Iκ Bα expression was significantly elevated (Figure 2D, 2E).

### Serum cytokines indicating multiple organ injury and systemic inflammatory responses are reduced in TLR4 mutant mice after intestinal IR

**Figure 3:**
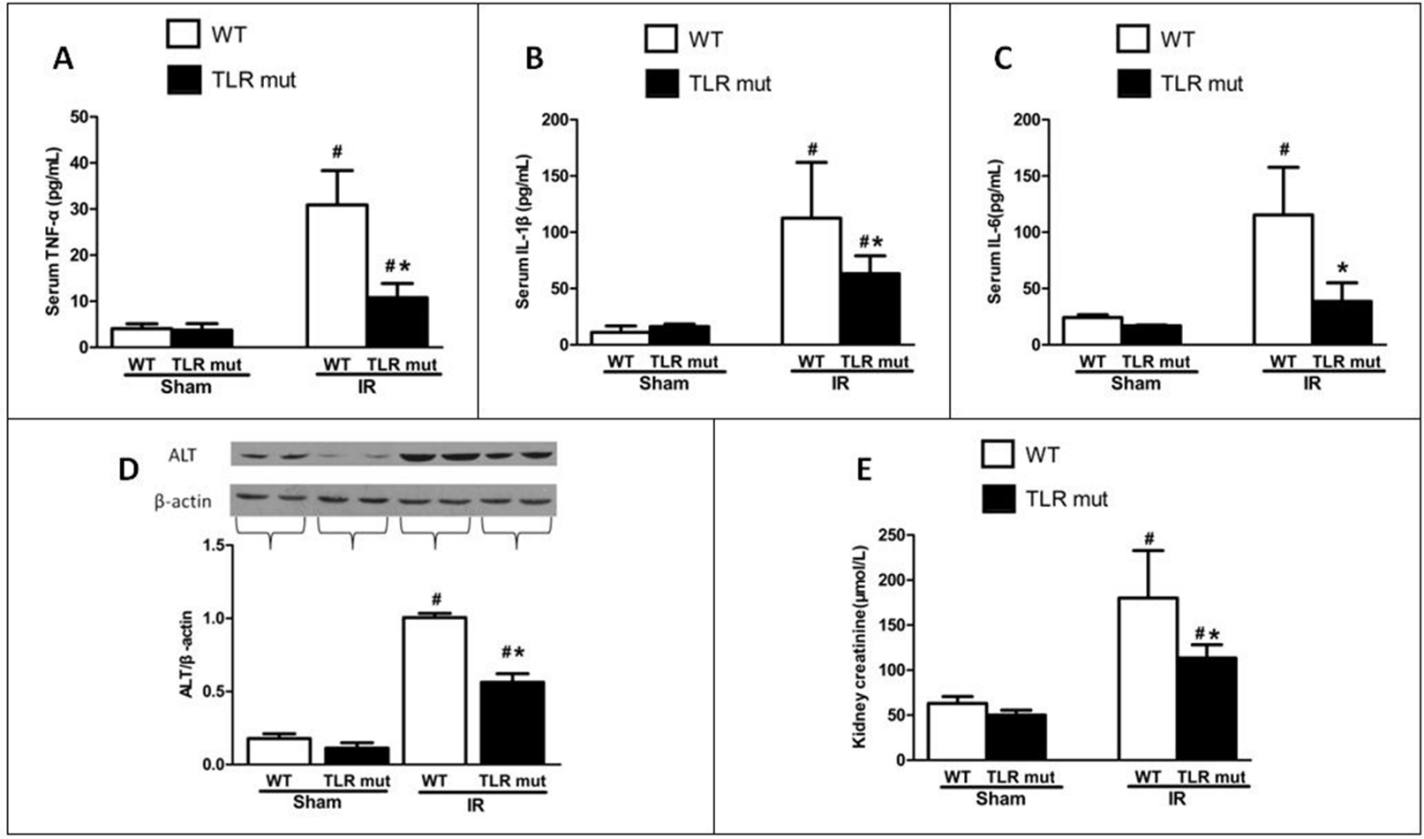
Effects of TLR4 mutant on the circulation and distant organs after intestinal IR WT mice and TLR4 mutant mice were sham-operated or subjected to 60 min of IR followed by 120 min reperfusion. Serum levels of (**A**) TNF-α, (**B**) IL-1β, and (**C**) IL-6 were measured by ELISA. Renal levels of (**E**) creatinine were measured by commercial kits. Hepatic expressions of (**D**) alanine aminotransferase (ALT) were determined by Western blot 120 min after reperfusion. Measurements were in triplicate. Bars denote the mean ± SD (n = 7/group). Data were compared by one-way ANOVA and LSD test. #*P* < 0.05 vs. WT sham and *P < 0.05 vs. WT IR.

Given the role of proinflammatory cytokines as mediators in the circulation between local intestinal injury and distant organ injuries, we next determined whether TLR4 mutant could alter the systemic inflammatory response by measuring blood markers of remote organ damage. Serum levels of TNF-α were significantly elevated by 6.6-fold in the WT IR group compared with the WT sham groups, whereas the TLR4 mutant significantly decreased the serum TNF-α concentration by 65.1% in comparison with the WT IR group (Figure 3A). Similarly, serum IL-1β and IL-6 levels were increased by 916.3% and 377.4% respectively in the WT IR group than in the WT sham group, whereas TLR4 mutant significantly abated IL-1β and IL-6 levels by 43.9% and 66.6%, respectively (Figure 3B, 3C).

### TLR4 mutant suppresses intestinal IR-induced ALI and ameliorates injury in other distant organs

**Figure 4:**
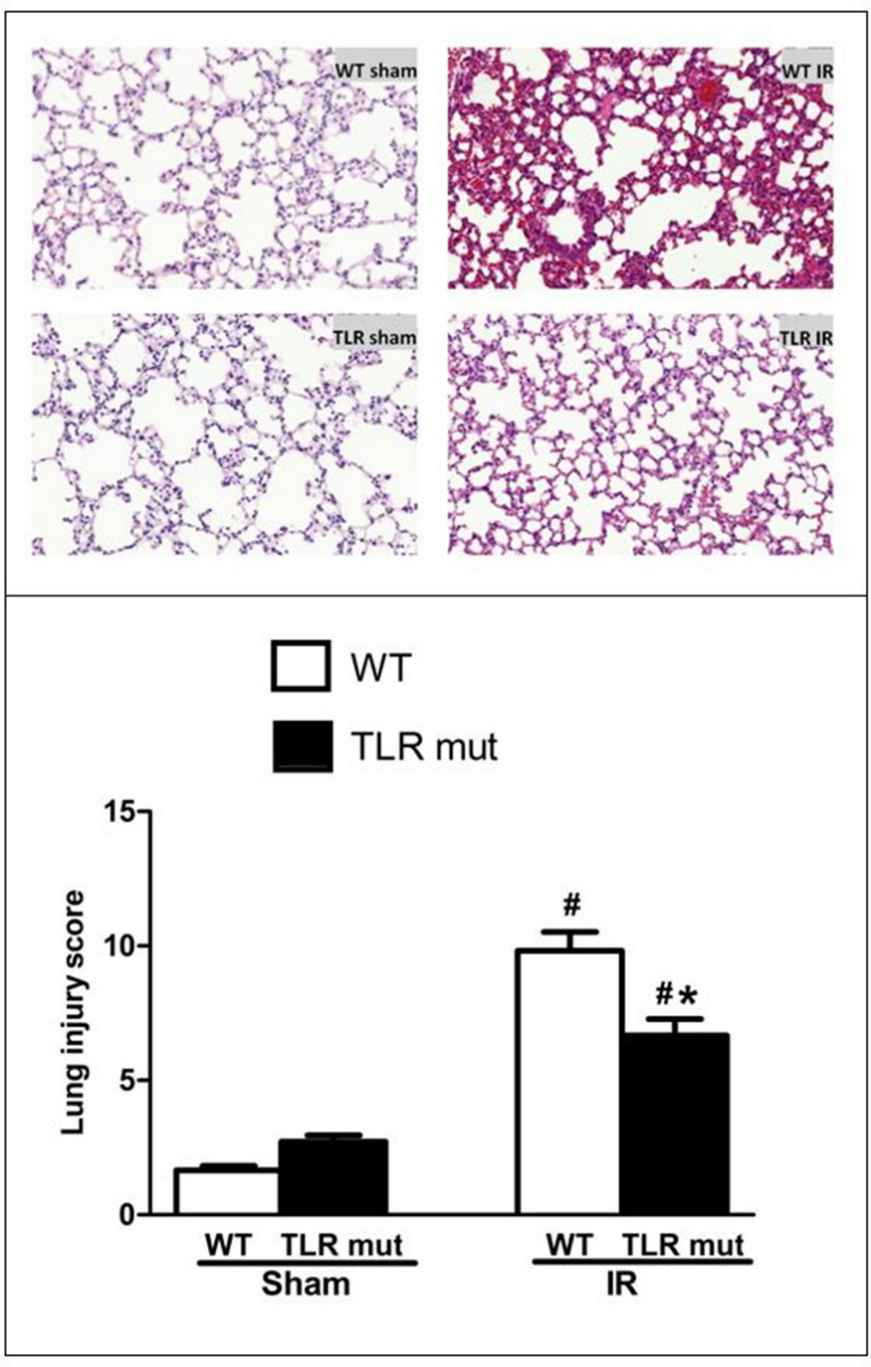
Alteration in the lung morphology after intestinal IR Representative micrographs at original 100 X magnification are shown. The SMA was occluded for 60 min, followed by 120 min reperfusion, to achieve intestinal IR. The lung tissues from the WT and TLR4 mutant mice submitted to IR or sham surgery were fixed and stained with HE. The lung injury was graded observing the presence of exudates, hyperemia/congestion, neutrophil infiltration, intra-alveolar hemorrhage/debris and cellular hyperplasia. Data were presented as mean ± SD (*n* = 7/group) and compared by one-way ANOVA and LSD test (#*P* < 0.05 vs. WT sham and *P < 0.05 vs. WT IR).

The lung is the most vulnerable organs after intestinal IR. Among the remote organ injuries affected by intestinal IR, the lung injury, specially resulting in ALI, predominantly leads to the high mortality induced by intestinal IR [25]. The morphological analysis of HE sections of the lung showed that in the sham group the lungs were presented with normal tissue-like appearance: there was neither exudate nor alveolar hemorrhage encountered in the lungs (Figure 4). Notably, in lung tissues of the WT IR group there were substantial alterations, including severe exudates, congestion, inflammatory cell infiltration, hemorrhage, and cellular hyperplasia, compared with sham group (Figure 4). TLR4 mutant prevented the pulmonary damage, with well aerated alveoli and few inflammatory cells observed, and the lung histological deterioration, as demonstrated by the pathological score, was significantly reduced by 32% in comparison with the WT IR group (Figure 4).

**Figure 5:**
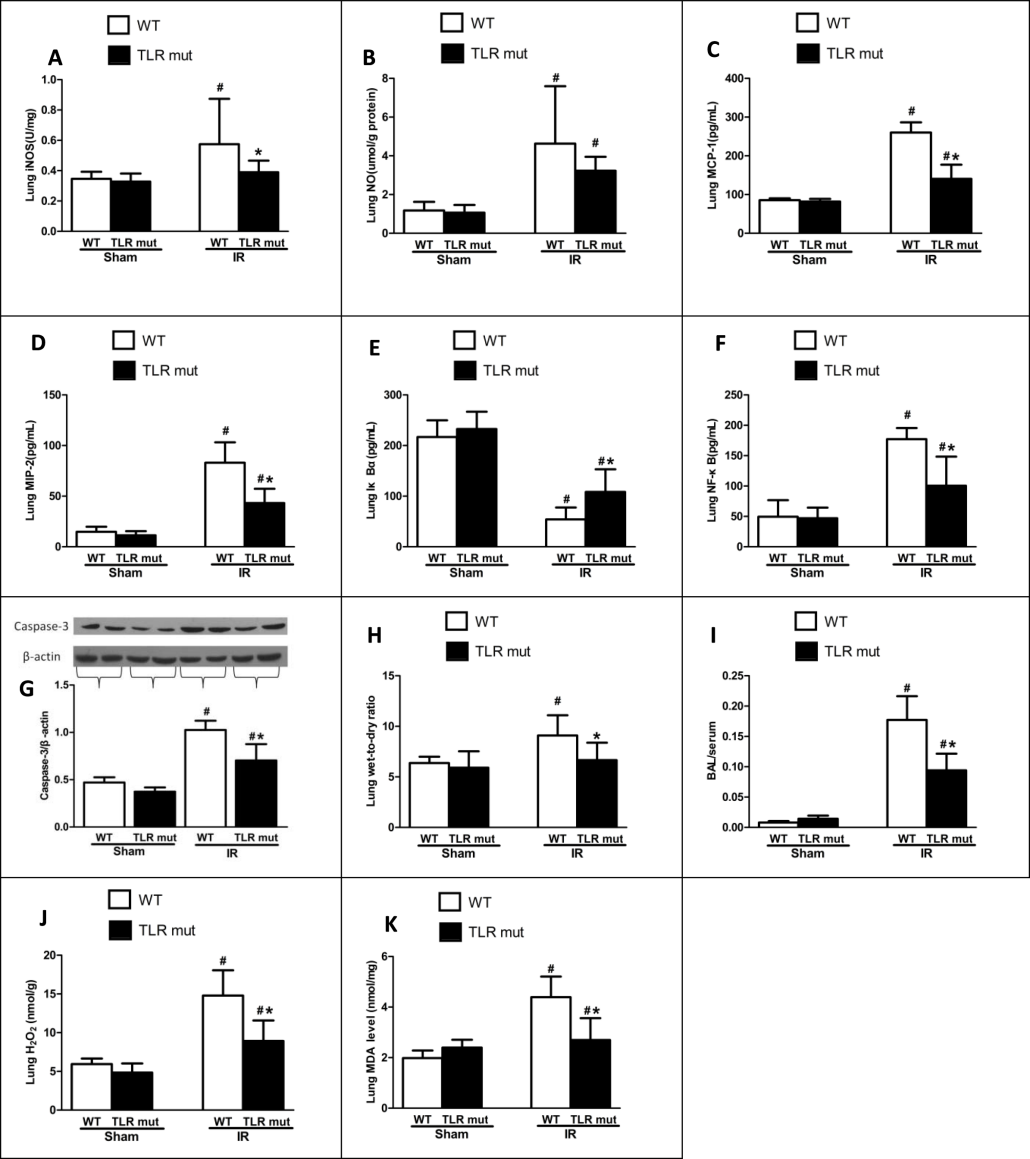
Lung cytokine expressions after intestinal IR WT mice or TLR4 mutant mice were submitted to 60 min IR followed by 120 min reperfusion (*n* = 7). After harvesting the tissues, cytokine expressions in the lungs were measure: (**A**) iNOS, and (**B**) NO by commercial kits; (**C**) MCP-1, (**D**) MIP-2, (**E**) IκBα and (**F**) NF-κB by ELISA; (**G**) Caspase-3 by Western blot with β-actin used as internal control for loading; (**H**) Wet-to-dry ratios; (**I**) Bronchoalveolar lavage (BAL) to serum protein levels; (**J**) Hydrogen peroxide (H2O2); (**K**) MDA. Results are representative of triplicate experiments from each group. Data were expressed as mean ± SD and compared by one-way ANOVA and LSD test. #*P* < 0.05 vs. WT sham and *P < 0.05 vs. WT IR.

In addition, the biochemical variables of the lungs tissues were measured. Consistent with the histological scores, although the oxidative stress markers of the lung tissues increased significantly after intestinal IR (iNOS by 0.66-fold, and NO by 2.92-fold) compared with the WT sham group, TLR4 mutant dramatically decreased the oxidative stress markers (by 32.2%, and 30.3%, respectively; Figure 5A, 5B). MCP-1 and MIP-2 were also significantly attenuated in the lung tissue of TLR4 mutant mice (Figure 5C, 5D). Apoptotic activities were measured by NF-κB, Iκ Bα, and caspase-3. The pulmonary expression of NF-κB protein was significantly down-regulated than the WT IR group, while the Iκ Bα expression was significantly up-regulated in the TLR4 mutant mice after intestinal IR (Figure 5E, 5F). Similarly, the elevated expression of caspase-3 in the lungs after IR was suppressed by 31.4% in the TLR4 mutant group (Figure 5G). In addition, the lung wet-to-dry ratios and BAL/serum protein ratios were significantly decreased in TLR4 mutant after IR, indicating improved lung permeability (Figure 5H, 5I). Lung hydrogen peroxide and MDA levels also exhibited similar suppressive trend (Figure 5J, 5K). Apart from the lung, others organs, including the kidney and liver, also showed decreased deterioration variables after IR, as demonstrated by significantly reduced levels of creatinine (by 37.0%) in the kidney and ALT (by 44.0%) in the liver (Figure 3D, 3E).

### Survival rate is improved in the TLR4 mutant mice after intestinal IR

**Figure 6:**
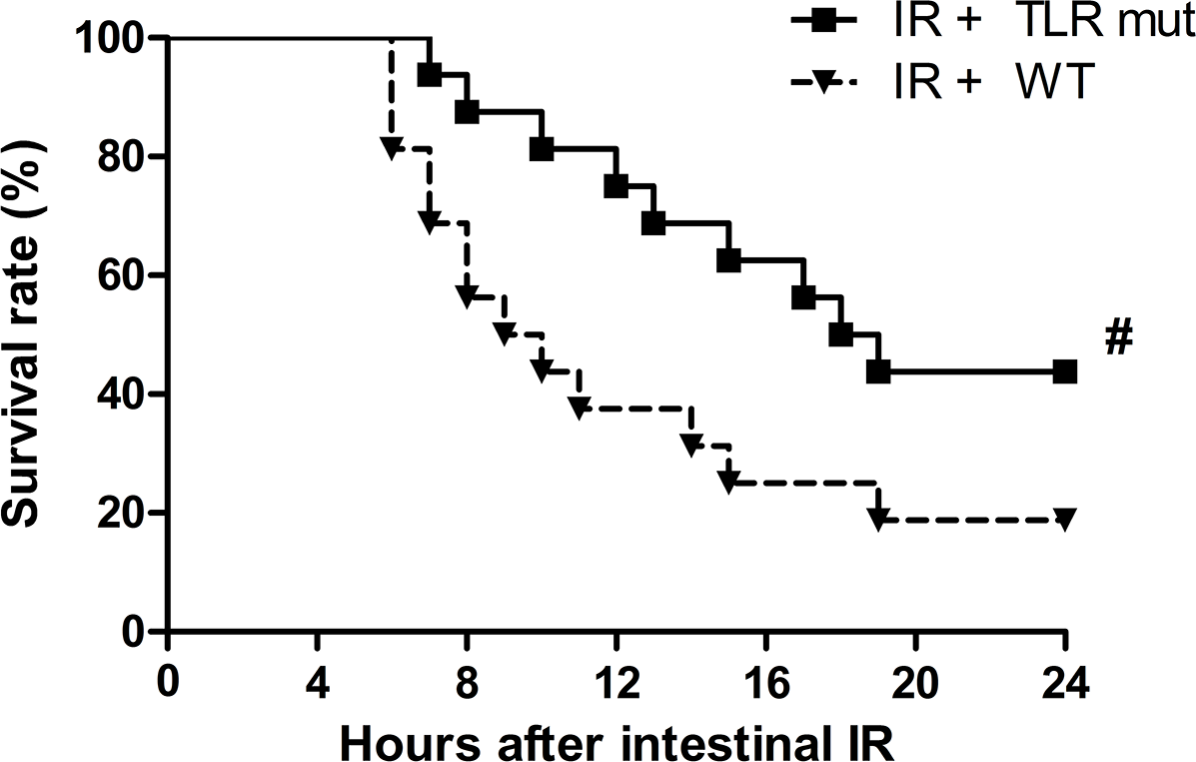
Survival benefit in TLR4 mutant mice after intestinal IR injury WT mice and TLR4 mutant mice were subjected to intestinal IR and then observed for 24 hours. Each point in the figure shows the mean survival rate at each time point. The survival rate was estimated by Kaplan-Meier method and compared by the log-rank test (*n* = 16/group). #*P* < 0.05 vs. WT mice with IR.

The above results so far demonstrated that TLR4 mutant was beneficial in alleviating multiple organ injuries after intestinal IR. We therefore conducted a 24-hour survival study to determine the long-term beneficial effect of TLR4 mutant in mice after intestinal IR. As shows in Figure 6, the earliest time point of death in mice was 6 and 7 hours after intestinal IR in the WT and TLR4 mutant group, respectively. Seven of the 16 mice in the TLR4 mutant group were still alive 24 hours after IR (median survival time: 18.0 hours; 95% confidence interval: 14.1–21.9; Figure 6). In the WT group, 13 of the 16 mice died within 24 hours (median survival time: 9.0 hours; 95% confidence interval: 5.1–12.9; Figure 6). The overall survival rate in the TLR4 mutant group was significantly higher than that of the WT group after gut IR (Figure 6; 43.8% vs.18.8%).

## DISCUSSION

In the current study, we have shown that intestinal IR caused devastation, not only locally in the gut but also remotely on the lung. These injuries were demonstrated by worsened intestinal and lung morphology and increased local and circulating inflammatory cytokine production consistent with pathological evaluations. TLR4 mutant attenuated local intestinal injuries, systemic inflammatory responses, and ALI after intestinal IR, ultimately contributing to improvement of survival in mice. Furthermore, the inflammatory injuries were mediated via NF-κB and caspase-3 pathways. Since TLR4 mutant mice showed a dramatically dampened ALI and inflammation, this experiment may provide further evidence for the essential role of TLR4 in IR-induced distant organ injuries.

ALI is one of the most important components of the multiple organ dysfunction syndromes triggered by intestinal IR injury and serves to be a leading cause of death in critically ill patients in ICU with the high mortality of approximately 40% [9]. Although the exact pathogenesis of ALI remains complex and poorly understood, yet there is ample evidence to support the gut hypothesis of multiple organ dysfunction syndromes [26], in which the damage of intestinal mucosal barrier after IR causes the translocation of bacteria with release of endotoxin into the systemic circulation, leading to systemic inflammatory response and activation of neutrophil infiltrations. The activated inflammatory cytokines and leukocytes result in over-production of reactive oxygen species and therefore subsequent distant organ injuries, including the development of ALI. TLR4 is responsible for pathogen recognition and activation of the innate immune system. It is able to recognize endogenous ligands, such as low-density lipoprotein and heat shock proteins, and exogenous ligands, such as the lipopolysaccharide, a component present in many gram-negative bacteria and select gram-positive bacteria [27]. Previous reports indicate that TLR4 is involved in organic IR and TLR4 knock-down can alleviate the organ damages [28, 29]. Consistent with previous reports, our study shows that TLR4 mutant mice exhibit decreased gut and lung injuries after intestinal IR.

Secondary inflammatory responses are vital in the process of ALI after IR, and the production of inflammatory mediators are dramatically increased during IR. There is abundant evidence suggesting that TNF-α, IL-1β, and IL-6 are pivotal mediators in intestinal IR and are regarded as molecular markers of inflammatory response in both intestine and lung after intestinal IR [30, 31]. Thus, strategies aiming at modulating the pivotal markers in activated inflammatory responses have been reported in many studies. A recent study indicates that TLR4 can mediate inflammatory response in alveolar macrophages [32]. Consistent with the reported significance of TLR4, we showed that TLR4 knock-down could lead to decreased expression of inflammatory cytokines. In our study, increased levels TNF-α, IL-1β, and IL-6 were observed in the mice model of intestinal IR injury, while TLR4 knock-down decreased the serum and tissue level of TNF-α, IL-1β, and IL-6, indicating that TLR4 knock-down inhibited inflammatory responses in the intestine and circulation and maintained a higher level of anti-inflammatory activity in mice with IR injury than in WT mice.

The exaggerated oxidative reaction and accumulated oxidants are also considered to play a critical role in the pathological mechanism of IR. Of the free radicals generated in IR, NO is of great importance. Overproduction of NO through activation of inducible NO synthase (iNOS) contributes to the damage after IR, whereas NO produced through constitutive NO synthase is beneficial and protective for its vasodilatory effects in smooth muscle cells. Previous researches indicate that iNOS inhibition prevented formation of excessive reactive nitrogen species and attenuated acute lung injury induced by combined burn and smoke inhalation [33]. Apart from its role in the lung, Chen *et al*. [34] reported that selective inhibition of iNOS ameliorates acute kidney injury in a rat model of myocardial IR. The results in our study were consistent with that of the previous reports. iNOS expression in the lung were elevated after intestinal IR and TLR4 knock-down blunted iNOS and NO expression in the lung tissue. The down-regulated expression of iNOS by TLR4 knock-down demonstrates its anti-oxidative function that may be a potential target for treatment in the future.

NF-κB is a widely-acknowledged transcriptional factor regulating the genes responsible for cellular stress and immune reactions [35]. Under physiological conditions, NF-κB is combined with the inhibitory κB (Iκ B), an inhibitory unit in the cytoplasm, to form an inactive state. The Iκ B consists of many subtypes, such as Iκ Bα, Iκ Bβ, Iκ Bε and Bcl3. When a multitude of extracellular signals transmit to the cell, NF-κB is released from the cytoplasm by phosphorylating its kinase Iκ B complex at the 32 and 26 position of serine, and enters the nucleus to regulate many gene expressions, including TNF-α, IL-6, and ICAM-1. Therefore, the interaction between NF-κB and Iκ Bα is the pivotal process of cell stress challenges [36]. In this study, the NF-κB expressions in the intestine and lung tissues were significantly ameliorated in the TLR4 mutant mice after IR than the WT mice, and the corresponding Iκ Bα expressions were up-regulated. This demonstrates that TLR4 knock-down is protective against IR injury in the local intestine and the distal lung organ through the NF-κB pathway. Since NF-κB is reported to be a major route for apoptosis in a variety of cell types including the intestinal epithelial cells [37] and pulmonary microvascular epithelial cells [38], we evaluated the expression of caspase-3, an “effector” protease in the apoptotic cascade, which is usually marked in intestinal IR. Consistent with our expectation, the results in our study showed that caspase-3 expression in the lung tissues was significantly decreased by TLR4 knock-down after IR. The possible anti-apoptotic effect of TLR4 knock-down may be associated with other evidences collected in the present study, such as reduced production of TNF-α and NO, thus inhibiting the activation of extrinsic apoptotic cascade.

There are several limitations in this study. Firstly, our apriori hypothesis is to some extent based on the gut hypothesis of systemic inflammatory responses and multiple organ dysfunction syndromes proposed by Deitch [26]. The bacteria translocation into the circulation that results from gut barrier dysfunction is central to the hypothesis. However, the failure to detect endotoxin in portal or systemic blood of critically ill patients makes the hypothesis arguable [39]. Nevertheless, there may be other non-microbial factors, such as the gut-lymph pathway [40], to link the gut and the distant organ injuries. In either case, systemic inflammatory responses mediate the multiple organ dysfunction syndromes. Considering that TLR4 knock-down reduced the systemic inflammation markers in the blood in this study, we have demonstrated that TLR4 knock-down is effective to protect the lung distally in the intestine IR-induced ALI model no matter it is by inhibiting bacteria translocation or through other pathways. Secondly, notwithstanding we found the caspase-3 expression was down-regulated in lung tissues after TLR4 knock-down, which may indicate a decreased apoptosis, the exact mechanism of TLR4 in protecting the lung from apoptosis during inflammation deserves further investigation. However, it has been reported that TNF-α and IL-6, two major inflammatory mediators of intestinal IR, can induce apoptosis through the activation of caspase-3 [41, 42]. In our experiment, the TNF-α and IL-6 production were significantly abrogated by TLR4 knock-down. When taken together, these findings imply that TLR4 knock-down dampens apoptosis in ALI, at least in part, through an indirect manner. Thirdly, the exact inflammatory cell type controlled by TLR4 is unknown in the lung. Since NF-κB signaling was disrupted by TLR4 in the lung tissue in this experiment, and NF-κB can modulate macrophages for releasing the inflammatory cytokines [43, 44], the alveolar macrophage is a plausible explanation for the anti-inflammatory effects of TLR4. However, further researches are needed to clarify this point.

In conclusion, the data presented in this study reveal that TLR4 knock-down significantly ameliorated the intestinal IR injury and ALI due in part to alleviating the inflammatory response and oxidative stress. Therefore, we postulate that agent blocking the TLR4 signaling pathway may be a clinically useful treatment in the future to relieve the tissue damage after intestinal IR. Further studies are required to clarify the question of whether blocking TLR4 can be applied to the clinical use for intestinal IR injuries.

## MATERIALS AND METHODS

### Animals and experimental grouping

C57BL/6 wild-type (WT) mice that were backcrossed 8 times and TLR4 homozygote mutant mice of the same genetic background of C57BL/6 were purchased from Charles River (Beijing, China). Young male mice (6–10-week-old; 20–30 g) were kept in sterile isolated polycarbonate cages with paper-chip bedding at optimum temperature (22 ± 2°C) and on a 12-h light/dark cycle. The mice were fed ad libitum with standard pellet diet and water to be acclimated for two weeks before the study. In the first set of experiment, mice were randomly allocated into four groups: sham + WT, sham + TLR4 mutant, IR + WT, and IR + TLR4 mutant. Each group consists of 7 mice. In the second set of experiment, another 32 mice were assigned into two groups to observe the survival rate over the course of 24 hours: IR + TLR4 mutant, and IR + WT (*n* = 16/group). This research was approved by the Institutional Animal Ethical Committee of Peking Union Medical College Hospital and the experimental protocol was in accordance with the Guide for the Care and Use of Medical Laboratory Animals (Ministry of Health, P.R. China, 1998).

### Animal model of intestinal ischemia reperfusion and acute lung injury

Twelve hours prior to the surgery, the mice were deprived of feeding but allowed free access to water only. At the beginning of the operation, sodium pentobarbital (50 mg/kg body weight) was injected intraperitoneally to induce anesthesia while the body temperature was sustained by means of a heating pad at around 36°C. Additional doses were given to maintain anesthesia if necessary. After the anesthesia, the mice were fixed in an operation pad at the supine position. The abdominal region was shaved and cleaned with iodine and alcohol solutions. Then the abdominal wall was opened through a 1.0-cm midline laparotomy, and the superior mesenteric artery (SMA) was carefully identified and occluded with an atraumatic microbulldog clamp to create ischemia for 60 min. Evidence of ischemia during the clamping period was confirmed by the existence of pulseless and pale color of the small intestine. After 1 hour of ischemia, the clamp was released, and three drops of normal saline were applied onto the SMA to facilitate reperfusion. Gross evidence of reperfusion was based on the return of pulses, the re-establishment of the pink color, and the enhanced intestinal peristalsis. Thus, the reperfusion was induced for 120 min. Sham groups were submitted to the same operation protocols, including the abdominal laparotomy and the isolation of SMA but with neither occlusion nor reperfusion. In addition, an observational experiment for survival rate over the course of 24 hours was carried out (*n* = 16/group).

### Biological specimen collections

At the end of the experiment, all mice were euthanized by giving an overdose of anesthetics drugs. The sternotomy was performed in the midline of the thorax to expose the heart and the lung. Blood samples were drawn by cardiac puncture in sterile syringes without anticoagulant and centrifuged (5000 g for 10 min at room temperature) to separate the serum. Portions of lung lobes (anterior, median and posterior lobe of the right lung; upper and lower lobe of the left lung) were harvested and every lung lobe was divided equally for both the histological examinations and the biochemical measurements. Then, the abdomen was further opened, and the whole small intestine was removed carefully. A segment of 3.0–4.0 cm small intestine locating 5.0 cm away from the terminal ileocecal valve was obtained. Each segment of intestine was cut equally into two parts: the first part was paraffin embedded for morphological analysis; the second part was dissected longitudinally on dry ice to expose the inner surface of the intestine, after being washed thoroughly with 0.9% normal saline, and dried with filter paper. Furthermore, portions of liver lobes (left, median and right lobes), and bilateral kidneys were also collected for investigation. The collected specimens were either: (1) fixed in 10% formalin and embedded in paraffin for histological analysis or (2) immediately snap frozen in liquid nitrogen and then stored at –80°C until biological assay.

### Hematoxylin and eosin staining of intestine and lung after IR injury

Morphological changes in the intestine and the lung were evaluated by hematoxylin and eosin (HE) staining. Generally, after paraffin embedding, 4-μm thick sections were cut in a freezing microtome, and then were fixed for 30 s and washed for 2 s. Then the sections were stained with hematoxylin at 60°C for 60 s, and then washed in running water for 10 s. Afterwards, the sections were incubated in 1% hydrochloric acid alcohol for 3 s, which was followed by a blue staining using promoting blue liquid for 10 s and then washed through running water for 30 s. Subsequently, 0.5% eosin was added to the sections for 60 s and the sections were washed using distilled water for 2 s. 80% ethyl alcohol for 2 s, 95% ethyl alcohol for 2 s, alcohol for 2 s, phenolic xylene for 3 s, xylene (I) for 3 s, and xylene (II) for 3 s were successively added to the sections. In the end, the sections were mounted using neutral balsam. Every lung lobe section was cut through the middle of the lobe so that every section included hilum to periphery, while every section of intestine was through the longitudinal line to continuously monitor the whole layer of mucosa.

### Pathological scoring system of injuries in the lung and intestine

The histological evaluation was conducted by two blinded experienced pathologists. In terms of the intestine, the injury degree was scored on a scale from 0 to 5 using a previously described scoring system for intestinal injury by Chiu *et al*. [45]. The Chiu's criteria consist of 5 subdivisions according to the changes of villus and gland of intestinal mucosa: Grade 0—Normal mucosal villi; Grade 1—Development of edema in sub-epithelial space, usually at the apex of the villus, often with capillary congestion; Grade 2—Extension of the sub-epithelial space with moderate lifting of epithelial layer from the lamina propria; Grade 3—Massive epithelial lifting down the sides of villi and a few tips may be denuded; Grade 4—Denuded villi with lamina propria and dilated capillaries exposed and increased cellularity of lamina propria may be noted; Grade 5—Digestion and disintegration of lamina propria and presence of hemorrhage and ulceration. The intestine injury score in the Chiu system from five sections was averaged to represent the grade for each animal. With respect to the lung section, the injury score was semi-quantitatively evaluated to the level of absent, mild, moderate, or severe injury (score 0–3, respectively) by observing the presence of exudates, hyperemia/congestion, neutrophil infiltration, intra-alveolar hemorrhage/debris and cellular hyperplasia. For example, for the presence of exudate: (0) no exudate (absent); (1) slight exudate from the alveolar walls (mild); (2) moderate edematous thickening of alveolar walls with occasional alveoli containing coagulated exudate fluid (moderate); (3) extensive occurrence of alveolar exudate (severe). For each animal, ten areas of the lung tissue section for each lung lobe were randomly selected within microscopic high power fields. The lung injury score assessed in each category for every individual mouse was the mean of the scores from the sections of the lungs examined, and total lung injury score was the sum of the scores in each categories. Slides of the lung and intestine sections were reviewed by the first investigator who did not know from which animal they were drawn. Then the slides were scored by the second investigator and any slide with a score difference of more than 1 was re-reviewed and assigned a consensus score.

### Quantification of organ injury cytokines

Frozen tissues samples of the intestine and lung were cut into 1–2 mm pieces, weighed and homogenized (100 mg tissue per mL of homogenization buffer). The homogenization buffer contained 1 mM PMSF, 1 μg/mL pepstatin A, 1 μg/mL aprotinin, and 1μg/mL leupeptin in PBS, pH 7.2, with 0.05% sodium azide and 0.5% Triton X 100. Samples were homogenized and subjected to one cycle of freeze-thaw, sonicated for 10 min and incubated at 4°C for one hour. Then the final homogenate was centrifuged at 120,000 g, and the supernatants were used for cytokine measurements. Tissue and serum levels of cytokines were measured by enzyme-linked immunosorbent assay (ELISA) kits specific for mouse TNF-α, IL-6, IL-1β, IκB-α, NF-κB p65, MIP-2, MCP-1, (Abcam, Cambridge, UK) according to the manufacture's instruction. Commercial assay kits were used to detect the level of NO, iNOS, hydrogen peroxide (H_2_O_2_), MDA, and creatinine concentrations (Abcam, Cambridge, UK). The cytokine contents were measured in triplicate and then averaged.

### Assessment of lung water content and lung permeability

Lung water content was determined by calculating the wet-to-dry ratios of the lung. In brief, the harvested lungs were weighed on an electronic scale to obtain the wet weight, and then dried in a thermostat oven at 60°C for 72 h before recording the dry weight. The wet lung mass divided by the dry lung mass represented the wet-to-dry ratio. Lung permeability was assessed from the ratio of protein concentration in bronchoalveolar lavage (BAL) to that in serum. To collect BAL, lungs were flushed with 0.9% normal saline in 2-ml volume four times, the recovery rate of which was approximately 80–90%. The protein content in the serum and BAL were measured by Coomassie brilliant blue staining method according to its protocol (Nanjing Jiancheng Institute, Nanjing, China).

### Western blot

Cellular protein was extracted from the frozen liver and lung tissues in a lysis buffer (50 mM Tris-HCl at pH 7.4, 150 mM NaCl, 0.1% sodium dodecyl sulfate [SDS], 0.1% sodium deoxycholate, 5 mM EDTA, 1% P-40 substitute [Nonidet], 1% polyethylene glycol tert-octylphenyl ether [Triton X-100], and 0.2% protease inhibitor cocktail [Abcam, Cambridge, UK]). Lysis buffer solubilized protein was denatured in a loading buffer (50 mM Tris-HCl at pH 6.8, 10% glycerol, 5% SDS, 5% mercaptoethanol, and 0.01% bromophenol blue) at 95°C for 5 minutes. The extracted 50 μg of tissue was fractionated on a Bis-Tris gel and transferred to a PVDF membrane filter by semidry electroblotting. To block the non-specific binding, the membranes were incubated with 5% skimmed milk in PBST. Then the membranes were incubated overnight at 4°C with a rabbit polyclonal caspase-3 antibody (dilution 1:80; Abcam, Cambridge, UK) or a rabbit polyclonal Alanine Transaminase antibody (dilution 1:1000; Abcam, Cambridge, UK) in PBST containing 0.5% bovine serum albumin (PBST-BSA). After washing 4 times in PBST, the membranes were then incubated with horseradish peroxidase-labeled goat anti-rabbit IgG antibody (dilution 1:3000; Abcam, Cambridge, UK) in PBST-BSA at room temperature for 1 h. Bands were detected using a chemiluminescent peroxidase substrate (ECL, Amersham, Beijing, China) and exposed on a radiograph film.

### Statistical analysis

The SPSS software (SPSS Inc., Chicago, IL, USA) was used for statistical analysis. Data were denoted as means ± standard deviation (SD) or percentage. The distribution analysis to confirm whether the data were normally distributed was performed by Shapiro-Wilk test. Differences between multiple groups were compared by one-way ANOVA with LSD to compare the difference between individual groups, assuming the data were conforming to normal distribution. If the data were not conforming to normal distribution, then the comparison of multiple groups was conducted by Kruskal-Wallis test, with Mann-Whitney *U* test to compare the difference between individual groups. The survival rate was analyzed by the Kaplan-Meier log-rank test. Statistical significance was set at a 2-tailed *P* value less than 0.05.
